# Influence of Estrogen Modulation on Glia Activation in a Murine Model of Parkinson's Disease

**DOI:** 10.3389/fnins.2017.00306

**Published:** 2017-05-31

**Authors:** Francesca Siani, Rosaria Greco, Giovanna Levandis, Cristina Ghezzi, Francesca Daviddi, Chiara Demartini, Elisabetta Vegeto, Marie-Thérèse Fuzzati-Armentero, Fabio Blandini

**Affiliations:** ^1^Laboratory of Functional Neurochemistry, Center for Research in Neurodegenerative Diseases, C. Mondino National Neurological InstitutePavia, Italy; ^2^Laboratory of Neurophysiology of Integrative Autonomic Systems, Headache Science Center, C. Mondino National Neurological InstitutePavia, Italy; ^3^Department of Pharmacological and Biomolecular Sciences, Center of Excellence on Neurodegenerative Diseases, University of MilanMilan, Italy

**Keywords:** Parkinson disease, 6-OHDA, estrogen, neuroinflammation, microglia polarization

## Abstract

Epidemiological data suggest a sexual dimorphism in Parkinson disease (PD), with women showing lower risk of developing PD. Vulnerability of the nigrostriatal pathway may be influenced by exposure to estrogenic stimulation throughout fertile life. To further address this issue, we analyzed the progression of nigrostriatal damage, microglia and astrocyte activation and microglia polarization triggered by intrastriatal injection of dopaminergic neurotoxin 6-hydroxydopamine (6-OHDA) in male, female and ovariectomized (OVX) mice, as well as in OVX mice supplemented with 17βestradiol (OVX+E). Animals were sacrificed at different time points following 6-OHDA injection and brain sections containing striatum and substantia nigra pars compacta (SNc) underwent immunohistochemistry for tyrosine hydroxylase (TH) (dopaminergic marker), immunofluorescence for IBA1 and GFAP (markers of microglia and astrocyte activation, respectively) and triple immunoflorescent to identify polarization of microglia toward the cytotoxic M1 (DAPI/IBA1/TNFα) or cytoprotective M2 (DAPI/IBA1/CD206) phenotype. SNc damage induced by 6-OHDA was significantly higher in OVX mice, as compared to all other experimental groups, at 7 and 14 days after surgery. Astrocyte activation was higher in OVX mice with respect the other experimental groups, at all time points. Microglial activation in the SNc was detected at earlier time points in male, female and OVX+E, while in OVX mice was detected at all time-points. Microglia polarization toward the M2, but not the M1, phenotype was detected in female and OVX+E mice, while the M1 phenotype was observed only in male and OVX mice. Our results support the protective effects of estrogens against nigrostriatal degeneration, suggesting that such effects may be mediated by an interaction with microglia, which tend to polarize preferentially toward an M2, cytoprotective phenotype in the presence of intense estrogenic stimulation.

## Introduction

Parkinson disease (PD) is one of the most common neurodegenerative disease of aging, neuropathologically characterized by progressive loss of dopaminergic neuron in the substantia nigra pars compacta (SNc) and depletion of dopamine in the striatum (Blandini et al., 2007). The pathogenetic mechanisms of PD are broadly described, with inflammation, apoptosis, aging and oxidative stress playing crucial roles (Blandini, 2013).

Epidemiological and biological data suggest the existence of a sexual dimorphism for PD: women show lower risk of developing PD, and their age at disease onset tends to be higher (Nitkowska et al., 2014). These differences are likely mediated by sex steroid hormones, estrogens in particular. Earlier observations have described an inverse correlation between estrogen levels and severity of PD symptoms (Currie et al., 2004), and the incidence and prevalence of PD is higher in postmenopausal than in premenopausal women of the same age (Currie et al., 2004; Ragonese et al., 2006a). Moreover, a significant correlation between age at PD onset and menopause has been reported, as well as an increased risk of PD in conditions causing reduced cumulative exposure to endogenous estrogens (early menopause, reduced fertile life length) (Ragonese et al., 2006a,b). Estrogen replacement therapy is associated with reduction of symptoms severity or lesser risk of developing PD (Tsang et al., 2001); by contrast the risk for PD increases in women with surgical menopause (Benedetti et al., 2001) or undergoing ovariectomy prior to menopause (Rocca et al., 2008). Thus, exposure to estrogen activity during lifetime may modify brain sensitivity to degeneration, influencing disease onset.

Estrogen neuroprotective effects have been widely reported in a number of neuronal cell systems, including the nigrostriatal dopaminergic neurons, via both genomic and non-genomic mechanisms (Morale et al., 2006). Several studies in animal models showed a positive influence of estrogens on neurodegeneration related to their anti-inflammatory and anti-apoptotic effects (Gillies and McArthur, 2010; Habib and Beyer, 2014; Villa et al., 2016). Effects on glial reactivity might be particularly relevant, as glial cell activation (microglial, in particular) may substantially contribute to progression of neuronal damage in PD (Armentero et al., 2006; Hirsch and Hunot, 2009; Whitton, 2010; Carta and Pisanu, 2013; Sanchez-Guajardo et al., 2013).

Activated microglia can be polarized and, depending on the modulatory effects of the microenvironment, driven toward different phenotypes: a cytotoxic, M1 or a neuroprotective, M2 phenotype (Hu et al., 2012; Blandini, 2013; Franco and Fernández-Suárez, 2015), two extremes of a range of intermediate phenotypes that microglia acquire in response to a myriad of physiological stimuli. Although mouse models are extensively used for macrophage/microglia research, translation to the human can be problematic, since human macrophage system remains poorly described. However, it is clear that the subsets between human being and mouse are similar but not identical (Ingersoll et al., 2010; Italiani and Boraschi, 2014; Zhang et al., 2017). Prolonged activation, as it seems to occur in PD, may promote the release of complement proteins, chemokines and cytokines, which activate M1 inflammatory responses with neurotoxic effects (Su and Federoff, 2014), thereby sustaining the neuronal degeneration in PD (Lull and Block, 2010). Their proliferation and activation, characterized by peculiar morphological changes and release of soluble factors (Colburn et al., 1997), is central to the maintenance of the immune homeostasis of the brain. Ovariectomy induces a decrease levels of estrogen receptor-α (ER-α) and an increase in the expression of neuroinflammatory markers, which are reduced by estrogen replacement therapy (Labandeira-Garcia et al., 2016).

Classic murine PD models, based on intracerebral infusion of 6-hydroxydopamine (6-OHDA) in rats or systemic administration of 1-methyl-4-phenyl-1,2,3,6-tetrahydropyridine (MPTP) to mice, have been used to explore the role of glial activation associated with nigrostriatal damage and the modulatory effects estrogens. In rats receiving an intrastriatal injection of 6-OHDA, which induces prompt damage of striatal terminals followed by delayed loss of SNc neurons (Domanskyi et al., 2011; Blandini and Armentero, 2012), microglia activation precedes cell loss in the SNc (Armentero et al., 2006) and shows a typical temporal profile, characterized by initial expression of the M2 phenotype progressively replaced by the M1 phenotype, as the nigral cell loss begins to manifest (Ambrosi et al., 2017). In MPTP-intoxicated mice, estrogen treatment have proven able to counteract the PD-like neuropathological alterations induced by the toxin by acting on dopaminergic neurons, either directly or through modulation of astroglial and microglial activation (Tripanichkul et al., 2006; Yadav et al., 2014). Through a slight modification of the classic 6-OHDA model (rat), Virgone-Carlotta et al. (2013) explored microglial activation in wildtype or transgenic C57BL/6N mice that received intrastriatal 6-OHDA. They demonstrated a direct crosstalk between SNc neurons and microglia that may trigger a microphagocytic activity toward dopaminergic neurons; also in this case, full microglia activation preceded maximum dopaminergic cell loss (Virgone-Carlotta et al., 2013).

In this study, we sought to investigate the potential estrogen modulation on glial reactivity during the progression of nigrostriatal degeneration in C57BL/6N mice exposed to unilateral intrastriatal injection of 6-OHDA. To examine the estrogen effects on the degree of glia activation and microglia polarization during the neurodegenerative process, we used male, female and ovariectomized (OVX) female mice treated with 17β-estradiol or placebo. The choice of using mice instead of rats, for the 6-OHDA model, was due to the necessity of testing the experimental design in animals (mice) suitable for subsequent studies in transgenic models.

## Materials and methods

### Animals

All experiments procedures were carried out in accordance with the European Communities Council Directives (2010/63/EEC; D.L., 27.01.1992, number 116) and the guidelines for animal experimentation approved by the Animal Care Committees of the University of Pavia. Maximum care was taken to limit the number of animals used in this study. Male and female C57BL/6N mice, 7–10 weeks old, were used (Charles River, Italy). Female mice were in proestrous stage, as determined by cytological assessment of vaginal lavage (McLean et al., 2012), when undergoing the experimental procedures. (OVX) C57BL/6N female mice (7 weeks) were also obtained directly from Charles River.

Animals were housed two per cage at the Centralized Animal Facility of the University of Pavia, under controlled conditions of light (12-h light/dark cycle) and temperature (20–22°C), with free access to food and water. Upon arrival, mice were left in the housing facilities for at least 1 week before the beginning of any experimental procedures. All animals received an estrogen-free diet (AIN93M Mucedola). One week after the ovariectomy, OVX mice were implanted with subcutaneous pellets (Innovative Research of America) that constantly released physiologic amounts of 17β-estradiol (17β-estradiol, 0.010 mg/day/mouse) (OVX+E) or placebo (Vegeto et al., 2006). The estrogen treatment in OVX+E mice was maintained throughout the experimental paradigm, until mice were sacrificed.

### Surgery

Mice were anesthetized with Equithesin (3 ml/kg i.p.) and placed in a stereotaxic frame (Stoelting, Wood Dale, IL,USA) completed with a mouse adaptor. Eight micrograms of 6-OHDA in 4 microliters of sterile saline solution containing 0.02% of ascorbic acid (Sigma, USA) were injected into the right striatum (+0.4 mm anterior, +1.8 lateral and −3.5 mm ventral with respect to bregma and dura) at 0.5 μL/min, using a Hamilton 10-μL syringe with a 26-gauge needle (Domanskyi et al., 2011; Virgone-Carlotta et al., 2013). After injection, the needle was left in place for 5 min before being retracted to allow complete diffusion of the medium, and wounds were clipped. In OVX and OVX+E groups, 6-OHDA was injected 2 weeks after the ovariectomy and 1 week after pellet implantation.

Male, female, OVX, and OVX+E mice were sacrificed 1, 2, 7, and 14 days after injection of the neurotoxin (*n* = 7 for each group, at each time point). Time points were selected in accordance to previously published data that demonstrate the most suitable time point to detect the onset of the neurodegenerative and neuroinflammatory processes (Virgone-Carlotta et al., 2013; Muñoz-Manchado et al., 2016).

The major advantage of using a neurotoxic PD model based on unilateral injection of 6-OHDA is the possibility to compare the lesioned side with the intact side, used as a negative control for histological analyses (Schober, 2004). Additionally, a substantial dopaminergic lesion can be prompted with 6-OHDA, whereas bilateral MPTP-induced lesions are less marked and sometimes more difficult to quantify. We thus exploited this experimental model of PD to clarify the role of estrogen on nigrostriatal vulnerability and to eventually extend these observations to transgenic models in future studies.

### Tissue processing

At sacrifice, animals were deeply anesthetized (Equithesin 3 ml/kg i.p.) and received a transcardiac perfusion of saline followed by 80 ml of ice-cold 4% paraformaldehyde (Merck VWR, Stockholm, Sweden). Brains were removed, post-fixed for 24 h in the same fixative and subsequently transferred in solutions of sucrose at increasing concentrations (up to 30%) over a 72 h period. Brains were frozen on dry ice, and stored at −80°C. Coronal brain sections containing the SNc and striatum were cut at 30 μm on a freezing sliding microtome (SM 200R, Leica, Milan; Italy) and stored at −20°C in a solution containing 30% ethylene glycol, 20% glycerol and 0.05 mol/L sodium phosphate buffer until use.

The nigrostriatal lesion was assessed by immunohistochemistry for the dopaminergic marker tyrosine hydroxylase (TH) on coronal sections of both SNc and striatum. Sections were processed with rabbit anti-TH primary antibody (AB152, Millipore, 1:500) and a biotinylated anti-rabbit IgG secondary antibody (Vector Laboratories, 1:500) and revealed using a commercial kit based on the avidin-biotin technique (Vectastain ABC Elite kit, Vector Laboratories). Reaction products were developed using nickel-intensified 3′-3′-diaminobenzedine tetra-hydrochloride (DAB Substrate Kit for Peroxidase, Vector Laboratories).

Assessment of neuroinflammation in the SNc was performed by glial and dopaminergic cells triple immunofluorescent labeling on sections containing SNc as follows: (1) sections were mounted on slides, dried at room temperature (RT) for 30 min and washed with phosphate buffered saline (PBS); (2) sections were blocked 1 h at RT in PBS containing 10% normal horse serum (NHS) and 0.3% Triton X-100 (TX-100) at RT; (3) sections were incubated overnight at 4°C in PBS/1% NHS/0.3% TX-100 containing a mixture of a mouse anti-TH antibody (MAB318, diluted 1:500, Chemicon) and either a goat anti-IBA1 antibody (microglial marker, diluted 1:300, Santa Cruz Biotechnology) or a rabbit anti-glial fibrillary acidic protein (GFAP; marker for astrocytes, diluted 1:300, Dako); (4) sections were rinsed in PBS and incubated 1 h at RT in PBS/1% NHS containing a mixture of Alexa Fluor 594 conjugated donkey anti-mouse IgG antibody (1:300) and Alexa Fluor 488 conjugated donkey anti goat IgG antibody (1:300) or Alexa Fluor 488 conjugated donkey anti rabbit IgG antibody (1:300) and Alexa Fluor 594 conjugated donkey anti mouse IgG. (1:300) (Molecular Probes, Carlsbad, CA, USA); (5) sections were rinsed in PBS and covered with Prolong containing DAPI to label cell nuclei.

Microglia polarization was assessed by triple immunofluorescent labeling on sections containing SNc as follows: (1) sections were mounted on slides, dried 30 min at RT and washed with PBS; (2) sections were blocked 1 h at RT in PBS containing 10% NHS and 0.3% TX-100 at RT; (3) sections were incubated overnight at 4°C in PBS/1% NHS/0.3% TX-100 containing a mixture of a goat anti-IBA1 antibody (marker for microglia, diluted 1:300, Santa Cruz Biotechnology) and either a rabbit anti TNFα or a rabbit anti CD206 antibody (both diluted 1:300, Novus and Santa Cruz Biotechnology, respectively); (4) sections were rinsed in PBS and incubated 1 h at RT in PBS/1% NHS containing a mixture of Alexa Fluor 488 donkey anti goat IgG antibody (1:300) and Alexa Fluor 594 donkey anti rabbit IgG antibody (1:300) e (5) sections were rinsed in PBS and covered with Prolong containing DAPI to label cell nuclei.

Negative immunofluorescent controls were performed by omitting primary antibodies from the otherwise regular immunolabeling protocol.

### Image analysis

#### Nigrostriatal degeneration

Image analysis was performed using an AxioSkop2 microscope connected to a computerized image analysis system (AxioCam MR5) equipped with a dedicated software (AxioVision Rel 4.2). The number of TH+ neurons in the SNc was counted bilaterally on every fifth section throughout the entire nucleus using the unbiased stereological optical fractionator method (Stereo Investigator System, Microbrightfield Inc.). Results were expressed as the percentage of TH+ cells in the lesioned SNc with respect to the intact side.

The 6-OHDA-induced loss of striatal dopaminergic terminals was evaluated by considering the striatal volume deprived of TH staining, as described previously (Blandini et al., 2007). Briefly, both the entire striatal area and the lesioned area were measured in every fourth section, throughout the whole rostro-caudal extension of the striatum.

#### Astroglia and microglia activation

The evaluation of the activation of astroglia and microglia within the SNc was determined by a qualitative analysis, observing SNc sections stained for GFAP or IBA1 at the microscope under medium magnification (20X). This analysis provides 4 activation stages, characterized by different morphological features and cell density (“0” = none; “+” = mild; “++” = moderate; “+++” = severe), according to criteria previously described (Ambrosi et al., 2017).

Moreover, cell count for microglia was performed by analyzing three different SNc sections, chosen according to their rostro-caudal coordinates. Cell density was assessed by counting IBA1+ cells from picture (0.04 mm^2^ frame, 40x magnification) taken from the same areas of the same SNc section. This analysis was performed only in OVX and OVX+E groups because in these experimental groups we observe significant differences in the progression of nigrostriatal degeneration and glia activation as compared to the other experimental groups.

### Statistical analysis

All values are expressed as mean ± SEM. Statistical evaluation of data was performed using a dedicated software (Prism 3 software, GraphPad Software). The differences between groups were analyzed by Two-way analysis of variance (ANOVA) followed by Bonferroni *post-hoc* test. Statistical significance was set at *p* < 0.05.

## Results

### Estrogen effect on 6-OHDA-induced nigrostriatal degeneration

**Figure 1:**
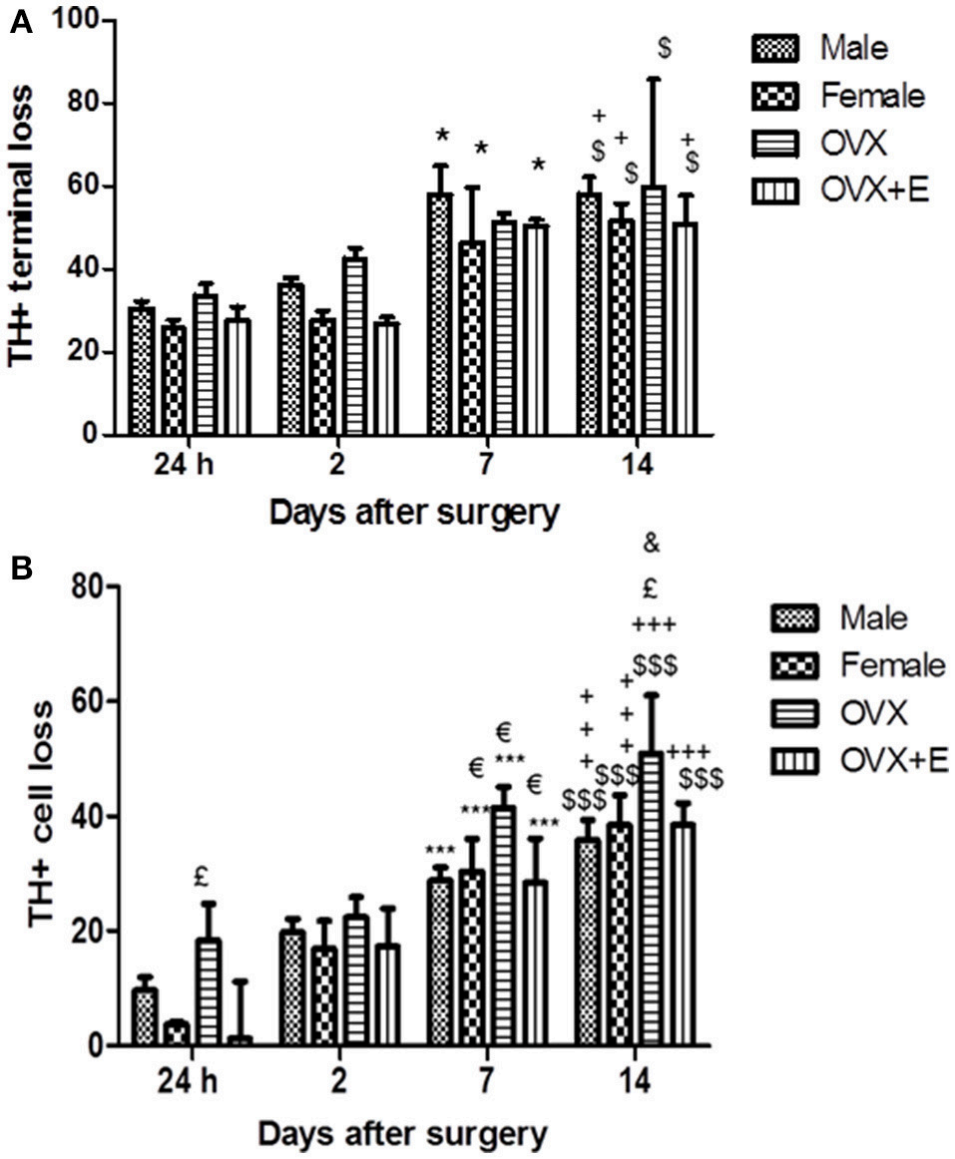
**(A)** Time course of striatal lesion. Histograms represent the percentage (%) of the striatal volume deprived of TH staining, compared to the entire striatum in the lesioned hemisphere. In the striatum, loss of TH+ terminals was present 24 h after 6-OHDA injection (25%) in the lesioned side, as compared to the contralateral hemisphere, and significantly increase at day 7 and 14 post-surgery with respect the early time points. Data are expressed as mean ± SEM. Two way ANOVA (*F* = 2.816) *post-hoc* Bonferroni test: ^$^*p* < 0.05 vs. 24 h; ^+^*p* < 0.05 vs. 2 days; ^*^p < 0.05 vs. 24 h. **(B)** Time course of TH-positive neurons loss in SNc. Histograms represent the loss (%) of TH-positive neurons of SNc in the lesioned hemisphere, compared with the SNc of the intact hemisphere. In the lesioned SNc, depletion of TH+ cell bodies was below 20% until day 2 and significantly increase at day 7 and 14 post-surgery with respect to the early time points. Data are expressed as mean ± SEM. Two way ANOVA (*F* = 1.195) *post-hoc* Bonferroni test: ^$$$^*p* < 0.001 vs. 24 h; ^+^^+^^+^*p* < 0.001 vs. 2 days; *p* < 0.05 vs. 2 days; ^£^*p* < 0.05 vs. OVX+E and female; ^&^*p* < 0.05 vs. male.

The intrastriatal infusion of 6-OHDA caused progressive loss of TH+ terminals and cell bodies in the ipsilateral nigrostriatal pathway, increasing significantly at 7 and 14 days after surgery, as compared to the early time points in all experimental groups (Figure 1). No cell loss was observed in the contralateral hemisphere. In the injected (right) striatum, loss of dopaminergic terminals stabilized at day 14 post-injection (Figure 1A). Male and OVX mice showed slightly higher levels of striatal damage compared to female and OVX+E mice, at all time points, although the difference did not reach statistical significance.

The loss of dopaminergic neurons in the SNc was expressed as a reduction in the number of TH+ neurons in the SNc of the injected (right) hemisphere as compared to the SNc of the intact hemisphere. Progressive, time-dependent loss of TH+ neurons was detected in the SNc of all experimental groups. Cell loss in the SNc was significantly higher at 7 and 14 days after the 6-OHDA infusion, as compared to earlier time points (Figure 1B). No significant differences were observed between male and female mice in the extent of dopaminergic cell loss in the SNc. Dopaminergic cell loss was significantly increased in the right SNc of OVX mice 24 h, 7 and 14 days after surgery (Figure 1B), as compared to the other experimental groups.

Interestingly, treatment with 17β-estradiol (OVX+E mice) reduced the dopaminercic cell loss, as compared to OVX mice, with values remaining in the range of those observed in male and female mice. In keeping with the findings of Virgone-Carlotta et al. (2013), showing a 25 and 30% loss of dopaminergic neuron at 4 and 7 days post-6-OHDA, respectively, we observed a dopaminergic cell loss, in male and in female mice, of 30% at day 7 and 40% at day 14 post-6-OHDA.

### Estrogen effects on neuroinflammation in the SNc

#### Astrocytes

**Figure 2:**
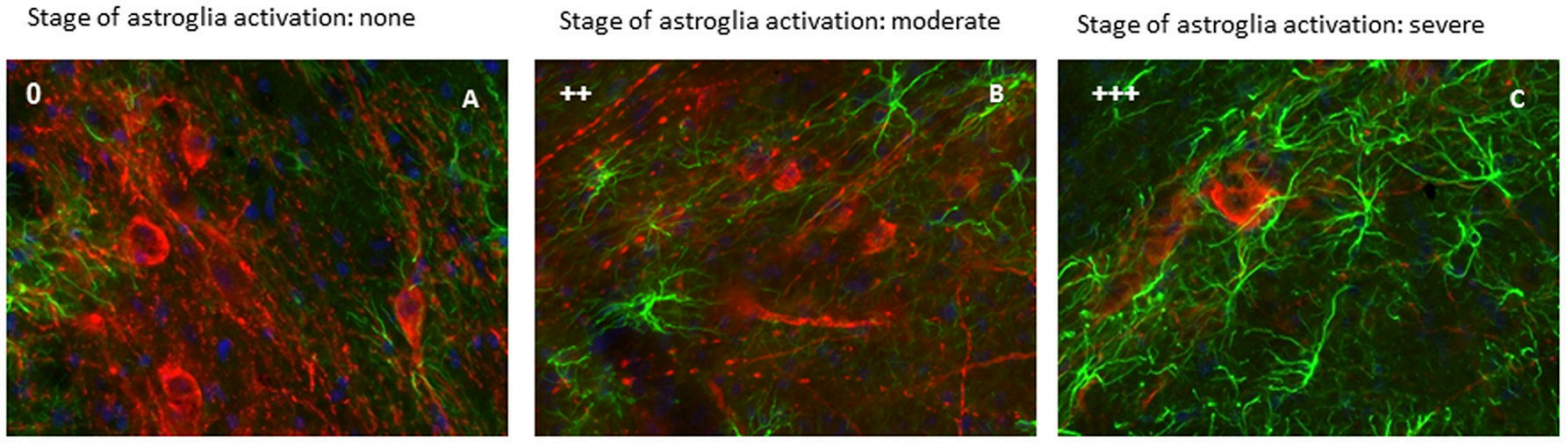
Activation of astrocytes was evaluated in the lesioned (right) SNc. This figure show a representative immunofluorescent images of the right SNc at different state of astroglia activation, determined by a qualitative analysis. Green signal: GFAP+ cells (astroglia); red signal: TH+ cells (dopaminergic neurons); blue signal: DAPI (nuclei). Astroglia activation stage 0 (A), astroglia activation stage ++ (B) and astroglia activation stage +++ (C) (scale bar 20 μm).

Astroglial and microglial activation in the SNc was assessed by qualitative morphological analysis and cell density evaluation (Tables 1, 2). No signs of astrocyte activation could be detected in the intact contralateral hemisphere of lesioned animals, with cells observed mainly around the SNc and no increase in cell density (Figure 2A).

**Table 1:** Time course of astrocyte activation in the substantia nigra pars compacta following 6-OHDA infusion.

**Experimental groups**	**1 day**	**2 days**	**7 days**	**14 days**
Males	0/+	+	+/++	++
Females	0/+	+	++	++
OVX	+	++	++/+++	++/+++
OVX+E	+	+	++	++

*The stages of astroglial activation were assessed according to the Colburn's scale. [0, resting stage; 0/+, astroglial cells are branched, but does not increase the cell density; +, astroglial cells are branched and decreases the intercellular space; +/++, intermediate state between stage 1 activation (+) and 2 (++); ++, astroglial cells lose the ramifications and increases the cell density]*.

**Table 2:** Time course of microglia activation in the substantia nigra pars compacta following 6-OHDA infusion.

**Experimental groups**	**1 day**	**2 days**	**7 days**	**14 days**
Males	+	+	0	0
Females	+	+	0	0
OVX	+/++	+	+	++
OVX+E	+/++	+	0/+	0/+

*The stages of microglia activation were assessed according to the Colburn's scale. [0, resting stage; 0/+, microglial cells are branched, but does not increase the cell density; +, microglial cells are branched and decreases the intercellular space; +/++, intermediate state between stage 1 activation (+) and 2 (++); ++, microglial cells lose the ramifications and increases the cell density]*.

In both male and female lesioned animals, a time-dependent increase in astrocyte activation, paralleling the progression of dopaminergic cell loss, was observed in the SNc of the injected hemisphere (Figures 2B,C). The intercellular space decreased both around and in the SNc, and astroglia displayed a clearly activated state, with increased GFAP expression, rounder cell bodies with thickening and reductions of projections, which evolved with time after 6-OHDA injection (Figures 2B,C; Table 1). Significant activation was observed 7 and 14 days after surgery as compared to earlier time points (Table 1).

Astrocyte activation was higher in OVX mice with respect the other experimental groups, at all time points. Notably, 17β-estradiol (OVX+E) clearly reduced the astrogliosis compared to that observed in OVX mice.

#### Microglia

No microglia activation was observed in the left SNc of lesioned animals. A time-dependent activation was observed in the SNc of the injected (right) hemisphere, which differed from that observed for astroglia (Table 2). In male and female lesioned mice, microglia activation increased immediately after 6-OHDA injection, with cells showing a slight increase in IBA1 expression and a reduction in the intracellular space. Activation peaked 2 days after the neurotoxic insult and then decreased, returning to a baseline, resting phenotype 7 and 14 days after surgery (Table 2).

**Figure 3:**
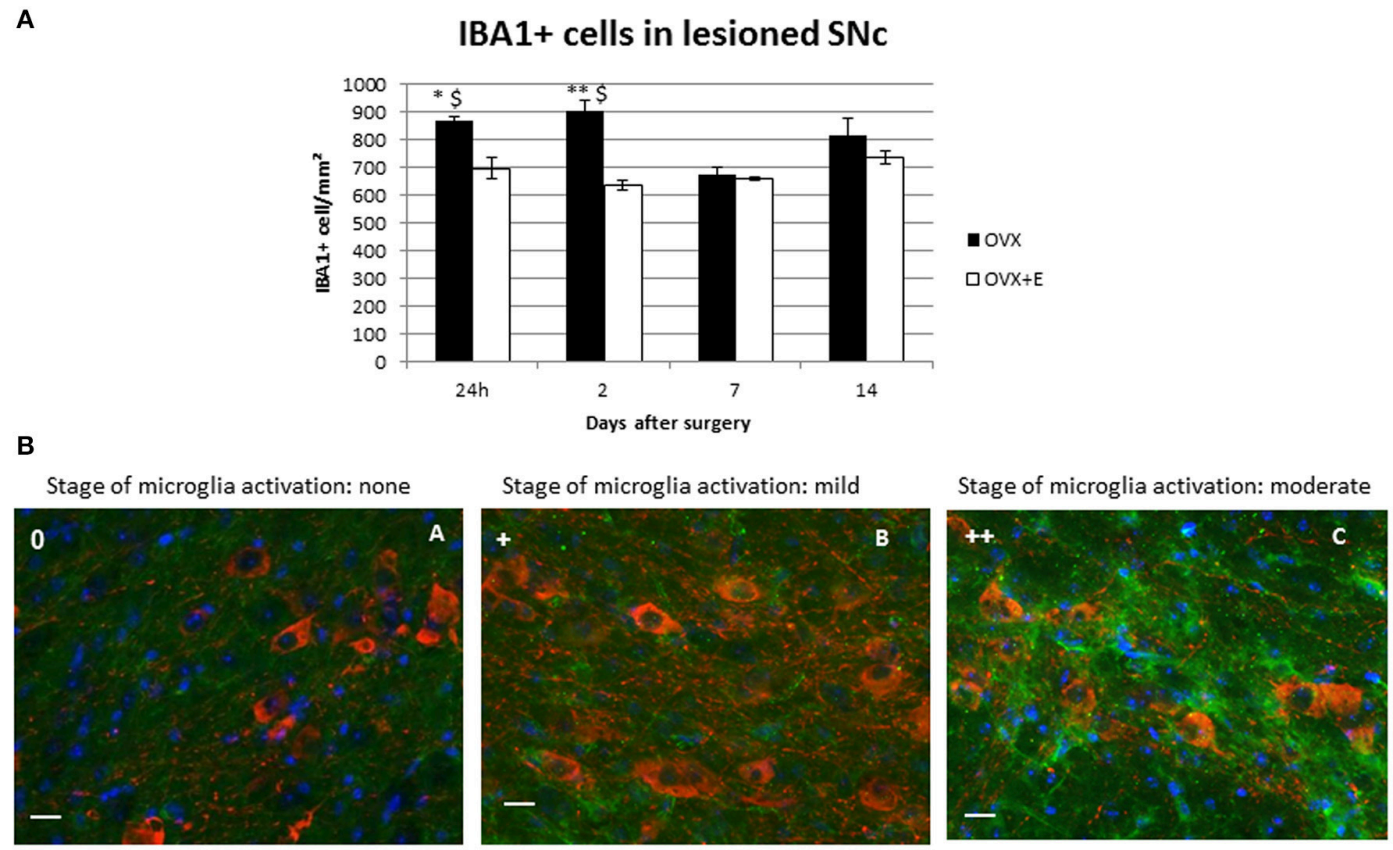
**(A)** Time-course of IBA1+ cell density in the lesioned (right) SNc. Approximately 800 cells/mm^2^ were detected in in the right SNc of OVX animals at all time points except for day 7 post-surgery. Results are expressed as mean ± SEM. Two way ANOVA (*F* = 4.22): ^*^p < 0.05 vs. OVX+E; ^**^p < 0.01 vs. OVX+E; ^$^*p* < 0.05 vs. OVX 7 days after surgery. **(B)** Representative immunofluorescent images of the right SNc at different state of microglia activation. Green signal: IBA1+ cells (microglia); red signal: TH+ cells (dopaminergic neurons); blue signal: DAPI (nuclei). Microglia activation stage 0 (A); microglia activation stage + (B); microglia activation stage ++ (C). Scale bar: 20 μm.

A higher activation state was observed at day 1 post-injection in OVX and OVX+E mice, compared to male and female mice, with microglia showing rounder bodies, less ramification and increased cell density both in and around the SNc of the injected hemisphere (Table 2, Figure 3B). This microgliosis extended throughout all time points considered in OVX mice.

Treatment with 17β-estradiol had no effect on the initial activation of microglia cells, but strongly shortened its duration, with microglia displaying an almost resting morphology 7 and 14 days after 6-OHDA injection in OVX+E mice. The pattern of microglia activation observed in OVX and OVX+E was associated with the increase of cell count. OVX lesioned mice show a significantly increased of IBA1+ cells density as compared to OVX+E, at 24 h and 2 days after surgery. Moreover, it was observed a significant increase of IBA1+cells in OVX group at 24 h and 2 days after surgery as compared to the later time points (Figure 3A).

#### Microglia polarization

**Figure 4:**
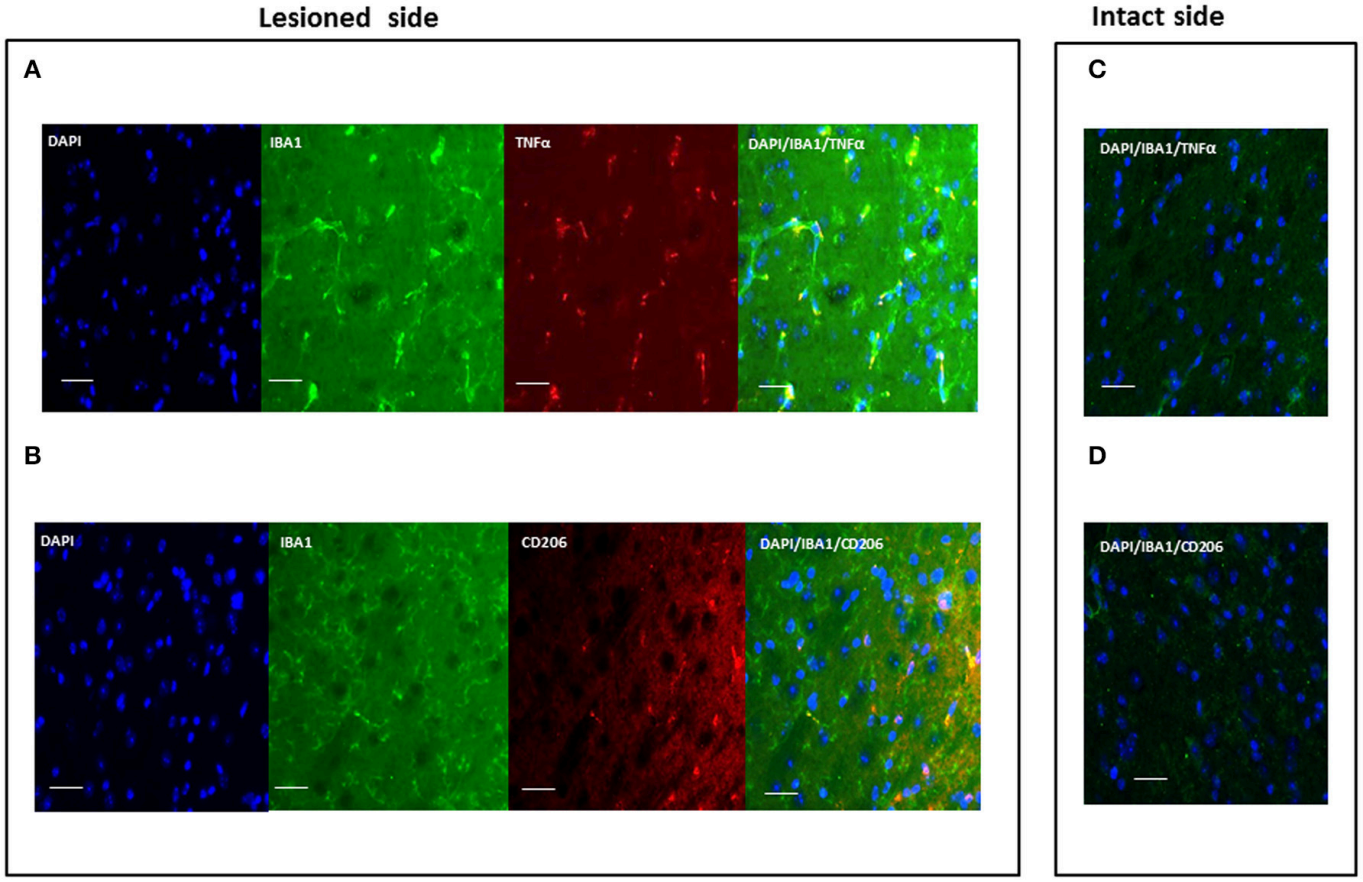
Representative immunofluorescent images of microglia polarization in the SNc. Images show a different phenotype of activated microglia polarization both evaluated in lesioned SNc: M1, cytotoxic **(A)** and M2, cytoprotective **(B)**; **(C,D)** are representative images of the intact side of SNc. Green signal: IBA1+ cells (microglia); red signal: TNFα (M1) or CD206 (M2); blue signal: DAPI (nuclei) (scale bar 20 μm).

Polarization of activated microglia was assessed using TNF-α and CD206 markers of microglia M1 (cytotoxic) and M2 (cytoprotective) phenotypes, respectively (Figure 4). These markers were not observed in resting microglia. No signs of activation were detected in the contralateral, intact hemisphere. Table 3 shows the evaluation of microglia polarization in the different time points after the neurotoxic injection, and Figure 4 shows a representative images of microglia polarization.

**Table 3:** Time course of microglia polarization in the substantia nigra pars compacta following 6-OHDA infusion.

**Experimental groups**	**1 day**	**2 days**	**7 days**	**14 days**
Males	CD206 (M2)	TNFα (Ml)	0	0
Females	CD206 (M2)	CD206 (M2)	0	0
OVX	CD206 (M2)	TNFα (Ml)	TNFα (Ml)	TNFα (Ml)
OVX+E	CD206 (M2)	CD206 (M2)	CD206 (M2)	CD206 (M2)

*All cells expressing the TNFα or CD206 antigen were also positive for microglial marker IBA1*.

Our data indicate that activated microglia cells displaying a cytoprotective phenotype (CD206+) were detected in the ipsilateral SNc 1 day after neurotoxin infusion (Table 3), in all experimental groups, independently of gender (male or female) or treatment (OVX or OVX+E). A sex-related difference could, however, be clearly observed 2 days after 6-OHDA injection. A switch from the M2 cytoprotective to the M1 cytotoxic phenotype, evidenced by the expression of TNF-α in activated microglia, was detected mainly in males, while a cytoprotective, CD206+ phenotype, was maintained in females.

A switch toward the M1 phenotype could also be detected in OVX mice at day 2, which was maintained throughout the experimental paradigm in activated microglia cells. Notably, treatment with 17β-estradiol (OVX+E mice) prevented the switch toward the M1 phenotype. Activated microglia maintained mainly CD206 expression, indicative of an M2, protective phenotype, at all time points in the ipsilateral the SNc of OVX+E mice (Table 3).

## Discussion

Many neurodegenerative diseases - including PD - show a sexual dimorphism, the risk of developing the disease being higher in men (Wooten et al., 2004). While it is now accepted that neuroinflammation plays a crucial role in the pathobiology of PD, the role of gender on onset and progression of PD and the effects of estrogens treatment on the neuroinflammatory response associated with PD are still unclear. Additionally, consistent evidence from experimental studies confirm the neuroprotective effects of estrogen in neurodegeneration. Incidence and prevalence of PD are slightly lower in premenopausal women, compared to men, but then increase after menopause (Currie et al., 2004; Ragonese et al., 2006a,b). Moreover, a reduced lifetime exposure of the nigrostriatal pathway to estrogens is associated with increased severity of PD symptoms, which can be further exacerbated after menopause (Cereda et al., 2013). It is described that women with a long fertile life tend to show a later onset of PD (Ragonese et al., 2004). Nevertheless, the association between the lack of estrogen observed after menopause and the progression of PD causes in women an higher risk of developing other complication, such as dyskinesia compared to men (Picillo et al., 2017). Clinical studies have been focused on the correlation between lifetime exposure to estrogens and risk of developing PD, as estrogen levels may influence the synthesis and release of dopamine, thereby modulating expression and function of dopamine receptors (Smith and Dahodwala, 2014). Clinical trial have demonstrated that estrogen replacement therapy is usually safe and well-tolerated at least for the short time treatment, while longer ones are necessary to define the effects of estrogens in PD and their eventually side effects. Moreover, other trials demonstrated mild efficacy of estrogens therapy in improving motor disability and motor fluctuations in PD post-menopausal women (Blanchet et al., 1999; Strijks et al., 1999; Tsang et al., 2000; Nicoletti et al., 2007; Parkinson Study Group POETRY Investigators, 2011). However, in a complex neurodegenerative disease, such as PD, it is difficult to translate the results from preclinical models to clinical studies, probably due to the presence of persistent stimuli, derived from endogenous different factors that contribute to increase neuroinflammation and consequently neuorodegeneration. Currently, the research in this field is still relevant because other studies are necessary to clarify the effective role of estrogens in PD, with the final goal to improve a tailored treatment for PD patients.

Experimental studies suggest a positive influence of estrogens on neurodegenerative diseases, possibly due to their anti-inflammatory, anti-apoptotic and cytoprotective effects (Habib and Beyer, 2014; Villa et al., 2016), in strict correlation with estrogen doses and formulations as well as timing and length of the dosing period (Shulman, 2002).

In this work, we have explored how manipulations of estrogen levels in females influence the vulnerability of the nigrostriatal pathway, focusing on the neuroinflammatory response associated with nigrostriatal degeneration. To pursue this goal, we have compared the nigrostriatal degeneration induced by intrastriatal infusion of 6-OHDA and changes in astrocyte and microglia activation/polarization in adult male and female mice, and OVX mice treated with estrogens or placebo.

Previous studies showed that smaller reductions of striatal dopamine are caused by neurotoxins when these are injected at the stage of the estrous cycle with maximal levels of estradiol (proestrus), compared to the diestrus (Yu and Liao, 2000; Datla et al., 2003; Dluzen and Horstink, 2003; Gillies et al., 2014). In our study, 6-OHDA infusion caused significant loss of dopaminergic terminals and neurons at 7 and 14 days after surgery, as compared to the earlier time points. We did not observed significant differences, in the striatal damage induced by 6-OHDA, between male and female mice, although the loss of dopaminergic terminals seemed to be more pronounced in male and OVX mice. By contrast, in previous studies, it was observed, that 2 weeks after administration of 1 μg 6-OHDA into the medial forebrain bundle, the depletion of both dopamine levels in the striatum and loss of dopaminergic neurons in the SNc was significantly greater in male rats compared to females (Murray et al., 2003; McArthur et al., 2007; Gillies et al., 2014). In particular, the progressive damage of dopaminergic cells over 5 weeks after lesion was also greater in 6-OHDA-treated male rats compared to females, demonstrating a sex difference. Probably, these sex differences are evident only after partial lesions (<60%) with moderate concentrations of 6-OHDA (Gillies and McArthur, 2010; Gillies et al., 2014). It is also possible that the different strain and neurotoxin regimen used in our study may account for this discrepancy.

Experimental models of neuroinflammation suggest that estrogens target primarily microglia (Vegeto et al., 2001, 2006; Johann and Beyer, 2013) and astroglia cells (Cerciat et al., 2010; De Marinis et al., 2013) in neurodegenerative diseases. Estrogen protect the nigrostriatal pathway in MPTP-intoxicated mice and the hormone response can be mediated by estrogen receptors express on microglia and astroglia cells, because estrogen receptors were not localized on TH+ terminals and neurons in the striatum and SNc, respectively (Shughrue, 2004; Almey et al., 2012).

In the present study, we found that OVX mice—which had undergone ovariectomy at the seventh week of life—showed greater loss of dopaminergic cell bodies in the SNc, as compared to the other experimental groups, at later time points (7 and 14 days post-6-OHDA). Remarkably, estrogen treatment reversed this change, OVX+E mice showing reduced loss of dopaminergic neurons in the SNc, 24 h, 7 and 14 days after 6-OHDA infusion, compared with OVX group, suggesting that estrogens treatment provide neuronal protection when it is given immediately after an ovariectomy. In addition, hormone deprivation worsens, while the constant exposure to estrogens relieves the damage induced by the neurotoxin in female mice. Probably, in OVX mice with estrogen deprived, the limited exposure to estrogenic stimulation during the fertile life caused an increased susceptibility of the nigrostriatal pathway to the neurotoxin. Accordingly, previous studies have shown that exposure to estrogens during the fertile life contributes to decrease the risk of PD (Ragonese et al., 2006a).

Immunoreactive microglia have been reported in both human brains and experimental models of PD, not only in the SNc, but also in the striatum, hippocampus and cortical areas implicated in disease (McGeer et al., 1988; Banati et al., 1998; Mirza et al., 2000). In addition, studies using the 6-OHDA model in rodents showed increased microgliosis associated with degeneration of dopaminergic neurons (Marinova-Mutafchieva et al., 2009). However, it is not yet clear whether the reactive microglia is a cause or effect of neuronal damage during disease progression, although studies support the idea that activation of microglia and thus inflammatory changes contribute to the disease processes.

In this study we showed that microglia is activated at earlier time points (1 and 2 days after 6-OHDA infusion) in the SNc of all experimental groups (Armentero et al., 2006; Virgone-Carlotta et al., 2013; Haas et al., 2016), but in male and female mice, then starts receding at day 7 and disappears at the latest time point (day 14). Here we also observed that IBA1+ cell density and activation is increased in OVX mice at different time points (24 h and 2 days after surgery) as compared to OVX+E mice and microglia activation, in OVX mice, remained elevated at all time points. We demonstrate that estrogen treatment restored the profile of microglia activation observed in male and female mice, confirming the dampening effects of estrogens on microglia activation.

Microglia can be polarized toward different phenotypes: cytotoxic M1 and neuroprotective M2 (Hu et al., 2012; Blandini, 2013; Joers et al., 2016). Transient activation of microglia is generally neuroprotective, but a chronic reactivity of these cells concur to the release of different cytotoxic molecules, such as reactive oxygen species, chemokines and pro-inflammatory cytokines (Su and Federoff, 2014), which play a pivotal role in PD neurodegeneration (Lull and Block, 2010; Perego et al., 2011). In this study we observed that the changes in microglial activation in the experimental groups were associated with significant differences in the polarization patterns, in particular among OVX mice and OVX mice treated with estrogen.

Our results demonstrate that in all experimental groups at 1 day after surgery microglial cells are polarized toward a CD206+(M2) phenotype, suggesting that the prevalent response of microglia in the early phases of neurotoxicity corresponds to a neuroprotective function. Nevertheless, 2 days after 6-OHDA injection microglia remained polarized toward the M2 phenotype only in female mice and OVX treated with estrogens. By contrast, in OVX mice we observed a prevalence of TNF-α+ microglia, compared to the other experimental groups, 14 days after surgery. Microglia activation polarized toward M1 phenotype was associated with a strong neuronal damage in OVX mice. By contrast OVX+E mice mainly showed a M2 phenotype and a significant reduction of damage in SNc. Additionally, the number of IBA1+ cells is significantly increase in OVX mice injected with placebo as compared to OVX mice treated with estrogen at 1 and 2 days after 6-OHDA infusion. These results strongly support that estrogen triggers pro-resolution effects on microglia, corroborating existing evidence obtained in cellular systems (Villa et al., 2015). Furthermore, these data allow to further strengthen the hypothesis that M2 polarized microglia participates in neuroprotection (Hu et al., 2012).

As for the astrocytic response to 6-OHDA, astroglia activation manifested at later time-points in all experimental groups. In OVX mice, astroglial activation showed the same pattern observed in intact mice, but was more intense, as compared to the other experimental groups, and decreased in OVX+E mice. In keeping with our observation, other studies have reported mild astroglial activation in the first week post-6-OHDA infusion, followed by more intense activation in the second week (Ambrosi et al., 2017). Astrocytes may either counteract or favor the neurodegenereative process, depending on the molecules released into the extracellular space and, consequently, the microenvironment shared with neurons (Johann and Beyer, 2013). Astrocytes can be directly activated by cytokines, such as TNF-α and IL-1β, released from microglia, thus leading to the production of free radicals and increasing the neuronal damage (Johann and Beyer, 2013). Changes in the microglia pattern of activation and polarization associated with ovariectomy, and restored after estrogenic treatment, may have therefore modulated astroglial reactivity. Furthermore, experiments in animal models have shown that the reduction of the astrocytes activation may also be linked to the reduction of neuronal damage, relative to a lower release of factors able to promote the astrogliosis (Ahaz-Fonseca et al., 2014). This effect could be mediated, once again, by interaction with ERs which could suffer a strengthening following infusion of 6-OHDA (Dang et al., 2011).

### Limitation of the study and future directions

It is known that ER activation in inflammatory cells plays a major role in the cytoprotective effect of estrogens and in the regulation of cerebral cytokines expression (Soucy et al., 2005). Additionally, in these cells, estrogens regulate protein expression involved both in inflammation and in remodeling of the extracellular matrix (Stygar et al., 2007). The present findings support that estrogen neuroprotection may be mediated by a direct effect on microglia, probably through the activation of the ER present on these cells (Vegeto et al., 2001, 2003). However, a limitation of the study resides in the lack of exploration of the selective ERs role and molecular mechanisms of estrogen-microglia signals underlying M2 polarization in the model used in this study. Consequently, future studies will be needed to define the influence of ERs on microglia activation related to 6-OHDA administration that induces neurodegeneration in ERs-KO mice.

## Conclusions

The identification of new therapeutic approaches able to stop or slow down the neuronal degeneration represents one of the major goals of current research in the PD field. Here, we have been demonstrated that estrogenic treatment is able to modify astroglia and microglia activation, microglia cell density, modulating the progression of nigrostriatal degeneration of PD. Future studies will be conduct for the development of “estrogen-like compounds” able to maximize the neuroprotective effect and, at the same time, to reduce the undesirable effects.

## Author contributions

Study conception and design: FB, MF, and RG; Acquisition of data: FS, GL, CG, and FD; Analysis and interpretation of data: FS, CD, EV, and RG. All authors were involved in drafting the article or revising it critically for important intellectual content, and all authors approved the final version to be published. All authors agree to be accountable for all aspects of the work in ensuring that questions related to the accuracy or integrity of any part of the work are appropriately investigated and resolved, and declare to have confidence in the integrity of the contributions of their co-authors.

### Conflict of interest statement

The authors declare that the research was conducted in the absence of any commercial or financial relationships that could be construed as a potential conflict of interest.
